# Draft Genome Sequence of Idiomarina sp. Strain W-5T, Isolated from the Andaman Sea

**DOI:** 10.1128/MRA.01510-19

**Published:** 2020-01-30

**Authors:** Sushanta Deb, Subrata K. Das

**Affiliations:** a Department of Biotechnology, Institute of Life Sciences, Bhubaneswar, India; University of Southern California

## Abstract

Here, we report the draft genome sequence of *Idiomarina* sp. strain W-5T, which was isolated from the Andaman Sea. The genome encodes 2,207 proteins. The *Idiomarina* sp. strain W-5T whole-genome sequence will help us to understand the metabolic diversity in marine bacteria.

## ANNOUNCEMENT

Ivanova et al. (1) proposed the genus *Idiomarina*, and to date, the genus consists of 28 species (http://www.bacterio.net/idiomarina.html). Species of *Idiomarina* have been reported mostly from seawater and hypersaline habitats (2–4). A recent study on the genus *Idiomarina* demonstrated that trophic specialization leads to genomic reduction in free-living marine bacteria (5).

*Idiomarina* sp. strain W-5T, used in this study, was isolated from Andaman Sea water (coordinates 11°59′54″N, 92°58′32″E). Water samples were collected from a depth of 3.5 m and transported to the laboratory for the isolation of bacteria. Aliquots (100 μl) of the water samples were serially diluted using phosphate-buffered saline (PBS) and plated onto marine agar 2216 (MA; Difco, USA), and plates were incubated at 25°C for 4 days. Colonies that appeared on MA (Difco) plates were picked and purified by repeated streaking on the same medium as described earlier (6). *Idiomarina* sp. strain W-5T was identified by chemotaxonomic and 16S rRNA gene sequence analysis (6). For the isolation of genomic DNA, the bacterium was grown in marine broth 2216 (Difco) for 48 h. Grown cultures (1.5 ml) were dispensed in a 2-ml Eppendorf tube, centrifuged, and washed with phosphate-buffered saline (PBS). The cell pellet was resuspended with 180 μl of ATL buffer (QIAamp DNA minikit [Qiagen, Germany]). The remaining steps and procedures for the isolation of genomic DNA, library preparation, and sequencing with the Sequel sequencing system (Pacific Biosciences, USA) were described earlier (7).

Quality control of the sequence reads was performed using –correct and –trim parameters inbuilt in the Canu 1.3 program. *De novo* genome assembly of PacBio reads was performed with the Canu 1.3 assembler (https://github.com/marbl/canu/) (parameters: correct; p, bacteria; merylMemory, 15; batThreads, 12; stopOnLowCoverage, 100; genomeSize, 2.4 million) (8). The scaffolding was performed using the Single Molecular Integrative Scaffolding (SMIS) pipeline (https://github.com/fg6/smis) (parameters: score, 50; len, 2,000; step, 200; contig, 3,000; edge, 5). Finally, the gaps were filled with the help of PBJelly (parameters: minMatch, 8; minPctIdentity, 70; bestn, 1; nCandidates, 10; maxScore, 500; nproc, 8; noSplitSubreads) (9). Perl Script (https://github.com/tomdeman-bio/Sequence-scripts/blob/master/calc_N50_GC_genomesize.pl) was used to calculate the statistical elements of the assembled genome. A total of 645,069 PacBio reads were assembled, which generated a single scaffold with an input read coverage of 800-fold. The assembled scaffold was circularized using the Minimus2 circularization pipeline (https://github.com/sanger-pathogens/circlator/wiki/Minimus2-circularization-pipeline), and the overlapping sequences were trimmed at the scaffold ends (10). The sequence was rotated using the TBLASTN feature of the Unicycler assembler using the –existing_long_read_assembly parameter (https://github.com/rrwick/Unicycler) (11). The draft genome was annotated using the NCBI Prokaryotic Genome Annotation Pipeline (PGAP) version 4.9 (12).The draft genome is 2.41 Mb with a G+C content of 47.05% and contains 2,207 protein-coding sequences, 56 tRNAs, 4 5S rRNAs, 4 16S rRNAs, and 4 23S rRNAs.

### Data availability.

The whole-genome shotgun sequences of *Idiomarina* sp. strain W-5T (= LMG 29773 = JCM 31645) have been deposited in DDBJ/ENA/GenBank under the accession number CP032551. Data under the accession number SRR7825310 are available at the NCBI SRA database.
